# Zoledronate suppressed angiogenesis and osteogenesis by inhibiting osteoclasts formation and secretion of PDGF-BB

**DOI:** 10.1371/journal.pone.0179248

**Published:** 2017-06-08

**Authors:** Si-yong Gao, Guang-sen Zheng, Lin Wang, Yu-jie Liang, Si-en Zhang, Xiao-mei Lao, Kan Li, Gui-qing Liao

**Affiliations:** 1 Department of Oral and Maxillofacial Surgery, Guanghua School of Stomatology, Guangdong Provincial Key Laboratory, Sun Yat-Sen University, Guangzhou, China; 2 Department of Oral Implant, Guanghua School of Stomatology, Guangdong Provincial Key Laboratory, Sun Yat-Sen University, Guangzhou, China; Augusta University, UNITED STATES

## Abstract

**Purpose:**

Bisphosphonates related osteonecrosis of jaw (BRONJ) is a severe complication of systemic BPs administration, the mechanism of which is still unclarified. Recently, platelet-derived growth factor-BB (PDGF-BB) secreted by preosteoclasts was reported to promote angiogenesis and osteogenesis. This study aimed to clarify whether bisphosphonates suppressed preosteoclasts releasing PDGF-BB, and whether the suppression harmed coupling of angiogenesis and osteogenesis, which could contribute to BRONJ manifestation.

**Methods and results:**

Zoledronate significantly inhibited osteoclast formation by tartrate-resistant acid phosphatase (TRAP) staining and PDGF-BB secretion tested by ELISA. In line with decreasing secretion of PDGF-BB by preosteoclasts exposed to zoledronate, conditioned medium (CM) from the cells significantly induced less migration of endothelial progenitor cells (EPCs) and mesenchymal stem cells (MSCs) compared to CM from unexposed preosteoclasts. Meanwhile, angiogenic function of EPCs and osteoblastic differentiation of MSCs also declined when culturing with CM from preosteoclasts treated by zoledronate (PZ-CM), evidenced by tube formation assay of EPCs and alkaline phosphatase activity of MSCs. Western blot assay showed that the expression of VEGF in EPCs and OCN, RUNX2 in MSCs declined when culturing with PZ-CM compared to CM from preostoeclasts without exposure of zoledronate.

**Conclusion:**

Our study found that zoledronate was able to suppress preosteoclasts releasing PDGF-BB, resulting in suppression of angiogenesis and osteogenesis. Our study may partly contributed to the mechanism of BRONJ.

## Introduction

Bisphosphonates (BPs) are one kind of anti-resorptive medication that are widely used in the treatment of osteoporosis and bone metastasis of malignancy. Bisphosphonates especially nitrogen-containing bisphosphonates (NBPs), such as pamidronate, alendronate, zoledronate have been reported to cause osteonecrosis of jaw since 2003 [1, 2]. It is now clear that bisphosphonates inhibit bone resorption by being selectively attached to mineral surfaces in bone where they interfere with the action of the bone-resorbing osteoclasts. NBPs affect osteoclast activity and induce apoptosis by inhibiting farnesyl pyrophosphate synthase (FPPS). Inhibition of FPPS prevents the biosynthesis of isoprenoid compounds which play critical roles in cellular growth and differentiation, cytoskeletal reorganization, gene expression and membrane ruffling [3]. However, the mechanism of bisphosphonate-related osteonecrosis of the jaw (BRONJ) is still uncertain.

NBPs were reported to reduce circulating levels of vascular endothelial growth factor (VEGF) in metastatic breast cancer patients [4]. And NBPs directly interfered with all major steps of the angiogenic process in endothelial cells, such as cell migration, proliferation and tube formation via prenylation-dependent signaling pathways or RhoA and MAPK signaling [5, 6]. The theory that the anti-angiogenic effect of BPs leads to BRONJ was also well illustrated by in animal models [7, 8]. The numbers and connections of blood vessels in teeth extraction sockets were significantly decreased in BPs-treated animals compared to control ones. On the other hand, the inhibition of osteogenic function and normal bone remodeling may attribute to BRONJ. Bisphosphonates might cause profound inhibition of osteoclasts function, resulting to disruption of the collateral communication between osteoclasts and osteoblasts or just show direct toxicity and inhibitory function to osteoblasts, which are not clear so far [9, 10]. However, the direct inhibition of angiogenesis and osteogenesis caused by bisphosphonates was generally observed in a high concentration in vitro experiments, which ranged from 10 μM to 100 μM, up to 10–100 times as the peak concentrations in plasma of patients given systemic BPs administration [11, 12].

The latest researches found that osteogenesis and angiogenesis were coupled during bone tissue development, regulated mainly by platelet-derived growth factor-BB (PDGF-BB) secreted by preosteoclasts during its maturation [13, 14]. These studies revealed that preosteoclasts-releasing PDGF-BB could not only induce osteoblastic differentiation of mesenchymal stem cells (MSCs) and promote endothelial progenitor cells (EPCs) differentiating into mature endothelial cells in vitro, but also induce CD31^hi^Emcn^hi^ vessel subtype in coupling osteogenesis in vivo, which plays a role in bone tissue healing and development. Besides the toxic effects of BPs to mature osteoclasts, inhibition of osteoclast formation from monocyte linage cells of BPs was also found [15, 16]. Therefore, we hypothesized that BPs might suppress the coupling of osteogenesis and angiogenesis by inhibiting the preosteoclasts-derived PDGF-BB secretion.

In this study, macrophages/osteoclast precursor cells (OPCs) were exposed to zoledronate during osteoclast formation and conditioned media (CM) were collected for PDGF-BB detecting and co-cultured with endothelial progenitor cells (EPCs) and MSCs. Then the migration assay was made to measure the chemotaxis for EPCs and MSCs. The function of angiogenesis were determined by tube formation assay and osteogenesis were determined by alkaline phosphatase (ALP) assay and mineralization nodules formation. Finally, the expression of VEGF of EPCs and RUNX2, OCN of MSCs were tested by western blot assay.

## Materials and methods

### Ethic statements

This study was carried out in strict accordance with Institutional Animal Care and Use Committee (IACUC) of Sun Yat-sen University and was approved by Animal Ethical and Welfare Committee of Sun Yat-sen University (IACUC-DB-16-1211).

### Reagents

Zoledronate (ZOL, Sigma-Aldrich, Germany) was dissolved in sterile phosphate-buffered saline (PBS, Gibco, USA) with a stock solution at 100 mM and filter sterilized using a 0.22 μm filter (Merk Millipore, USA). ZOL solutions were stored at 4°C.

### Cells culture and characterization

Adult male C57BL/6 mice aged 6–8 weeks were primarily purchased from Animal House Center of Sun Yat-sen University, Guangzhou, China. Mice were sacrificed by cervical dislocation and femora and tibiae were thoroughly dissected from muscle under sterilized environment. Both epiphyses were removed and bone marrow was flushed with 25×g syringe containing alpha-Modified Eagle’s Medium (α-MEM, Gibco, USA) till the femora and tibiae turned white. Total mononuclear cells (MNCs) were isolated from the cell suspension using density gradient centrifugation with Histopaq-1083 (density 1.083 g/mL, Sigma-Aldrich). The femora and tibiae were reserved for cultivating MSCs.

Mononuclear cells were plated on Petri dishes (Corning, USA) overnight in α-MEM containing 10% fetal bovine serum (FBS, Gibco), 100 U/ml penicillin, 100 μg/ml streptomycin sulfate and 30 ng/ml M-CSF (Sino Biological Inc., China) at 37°C in a 5% CO_2_/95% air incubator. After discarding the adherent cells, floating cells were plated on a new Petri dish and became adherent after a 3-d culture and were used as osteoclast precursor cells (OPCs). Then OPCs were digested by Trypsin–EDTA solution (0.25% Trypsin, 0.5mM EDTA, Gbico) and seeded in 96-well plates (2×10^4^ cells per well) with additional 60 ng/ml RANKL (R&D Systems Inc. USA) to induce into osteoclasts [17]. The OPCs were identified by flow cytometry analysis of surface maker F4/80 (12–4801, monoclonal, PE-labeled, eBioscience Inc., USA) and preosteoclasts as well as osteoclasts were identified by Tartrate Resistant Acid Phosphatase staining (TRAP staining, Sigma-Aldrich).

For EPCs cultivation, MNCs mentioned above were plated on fibronectin-coated dishes at a density of 5×10^6^/cm^2^ and induced by EGM-2 MV BulletKit (LONZA, USA) which comprises endothelial basal medium (EBM) and EGM-2 MV SingleQuot Kit Suppl. & Growth Factors (recombinant human fibroblast growth factor-β [rhFGF-β], recombinant human epidermal growth factor [rhEGF], R3-insulin-like growth factor [IGF]-1, vascular endothelial growth factor [VEGF], ascorbic acid and GA-100) and 10% FBS. After 3 days, non-adherent cells were removed by washing with PBS twice and changed new medium every 3 days. After 21 days in culture, EPCs were identified by function of in vitro tube formation and immunofluorescence for both Dil- Ac-LDL (Invitrogen, USA) and FITC-UEA-1 (L9006, Sigma-Aldrich) [14].

The flushed femora and tibiae were cut into pieces with scissors and washed four times with PBS to remove the remained hematopoietic cells. After digested by collagenase II (Sigma-Aldrich) at 37°C for 1–2 h, bone chips were plated on Petri dishes containing OriCell^™^ Mouse Mesenchymal Stem Cell Growth Medium (Cyagen Biosciences Inc., USA). Fresh medium was renewed every 3 days. When Spindle-shape cell migrated out of bone chips and approximately reached 80–90% confluence, they were harvested with Trypsin–EDTA solution for 1–2 mins and seeded at a concentration of 1×10^5^ cells/cm^2^ in T25 flasks (Corning). Cells of the 5th passage were identified and checked for purity with osteogenic, adipogenic differentiation identification and flow cytometry of surface marker: Sca-1, CD44, CD45, CD34 (25-5981-81, 12-0441-81, 11-0451-81, 11-0341-81, monoclonal, anti-mouse, eBioscience Inc.) [18].

### Effect of ZOL on viability of OPCs, EPCs and MSCs

OPCs, EPCs and MSCs suspensions were respectively seeded into 96-well plates at a density of 5×10^3^ cells/100 μL/well and incubated at 37°C. Following a 24 h incubation period, cells were treated with different concentrations of ZOL (0–100 μM). The treated cells were subsequently incubated for different time intervals of 24, 48 and 72 h. Cell viability was determined by 2-(2-methoxy-4-nitrophenyl)-3-(4-nitrophenyl)-5-(2,4-disulfophenyl)-2H-tetrazolium, monosodium salt (WST-8) assay kit (CCK-8, Dojindo, Japan). Briefly, 100μL 10-fold dilution tetrazolium solution was added to each well 1 h in dark at 37°C. The absorbance at 450 nm was measured using a microplate reader [6]. The toxic effect of ZOL on viability was measured by IC50, which was calculated by software SPSS 20.0.

### Effect of ZOL on osteoclasts formation

It has previously been demonstrated that OPCs became preosteoclasts in the present of M-CSF and RANKL within 3 days and the PDGF-BB secretion peaks during 48–96 h [13, 19]. Based on the cell viability assay, different concentrations of ZOL were added to OPCs at the beginning of differentiation for 2 days and then changed new medium every two days until osteoclast formation was completed. TRAP staining was tested for identifying osteoclasts formation and the TRAP-negative nuclei were counterstained by DAPI (Beyotime Biotechnology, China). Osteoclast formation was quantified by osteoclast fusion index, defined as number of nuclei per one multinucleated osteoclast.

### Conditioned media preparation and Enzyme-linked immunosorbent assay (ELISA)

Different types of conditioned media were prepared and collected, which were stored at -80°C after a centrifugation at 2500 rpm for 15 min at 4°C. Conditioned media were collected every 24h during osteoclast formation and then tested by Enzyme-linked immunosorbent assay (ELISA, R&D Systems Inc., USA) to confirm that preosteoclasts release PDGF-BB. Conditioned medium from preosteoclasts (PO-CM) with serum were collected for co-culturing with EPCs and MSCs. Serum-free PO-CM were collected for migration experiments. After the preosteoclasts being treated by different concentrations of ZOL for two days and washed by PBS twice, the ZOL-free conditioned medium from preosteoclasts (PZ-CM) were collected and tested by ELISA for PDGF-BB, as well. Besides, conditioned medium from preosteoclasts added with the neutralizing antibodies for PDGF-BB (ab23914, polyclonal, Abcam, 1 μg/ml, USA) for two hours before collection (PA-CM). The PA-CM were collected for negative control to make sure the intermediary function of PDGF-BB and conditioned media from macrophages/OPCs (M-CM) were also obtained as negative control in parallel and applying the same protocol, respectively.

### Migration assay of EPCs and MSCs

We assessed cells migration in Transwell-24 well plates (Corning) with 8 μm pore filters. EPCs or MSCs were seeded in the upper chambers at a density of 1×10^4^ cells/well with serum-free α-MEM and the serum-free conditioned media were in the lower chambers. After 24 h, cells on the upper surface of each filter were removed with cotton swabs and cells on the lower chambers were fixed with 4% paraformaldehyde for 15 min and then stained with crystal violet (Sigma-Aldrich) for 30 min. Cells migrated through the pores to the lower surface were quantified by counting five random high power fields per well using a microscope (Zeiss) at ×100 magnification [6].

### In vitro tube formation assay of EPCs

Tube formation assay in vitro was made to explore whether conditioned media affected EPCs the capacity of vascular formation. For tube formation assay, Matrigel matrix (Corning) was thawed on ice overnight, and placed in a 96-well tissue culture plate at 37°C for 1 h to allow the matrix solution to solidify. EPCs were placed on the top of the solidified matrix solution with conditioned media mentioned above at a concentration of 2×10^4^ cells/well. Four hours later, five independent fields were assessed for each well, and qualification was made by using plugin “Angiogenesis Analyzer” for software imageJ. The average number of tubes/x100 field was determined [6].

### Assessment of osteogenesis of MSCs

MSCs were seeded in 6-well plate at a density of 5×10^5^/well containing OriCell^™^ Mouse Mesenchymal Stem Cell Growth Medium. After a 24 h incubation period, medium was changed to serum-containing conditioned media. After a 2-d culture, the cells were homogenized and assayed ALP activity using an Alkaline Phosphatase Assay Kit (Beyotime Biotechnology, China). After 3 weeks of culture, the cell matrix mineralization was tested by 0.1% of Alizarin Red S staining (Sigma-Aldrich) [10].

### Western blot analysis

MSCs were cultured with the conditioned media mentioned above for 48 h and EPCs were co-cultured with the conditioned media for 4h, as long as the tube formation assay. Then the cells were lysed in buffer and total protein contents were determined using the BCA protein Assay Kit (ComWin Biotech Co. Ltd, China). Equal amounts of protein were subjected to SDS-PAGE and transferred to PVDF membranes (Merk Millipore, USA). The membranes were blocked with 5% non-fat milk, probed with anti-VEGF (ABS82, polyclonal, Merk Millipore, USA), anti-PDGFRβ (MA5-15143, monoclonal, Thermo Fisher Scientific, USA) for EPCs and anti-RUNX2 (ab23981, polyclonal, Abcam, USA), anti-OCN (ab93876, polyclonal, Abcam), anti-PDGFRβ for MSCs. The results were normalized to the expression level of β-actin (ab3280, monoclonal, Abcam). Then the membranes were stained with horseradish peroxidase-coupled secondary antibodies. Protein bands were visualized using enhanced chemiluminescence (GE Healthcare Life Sciences, UK).

### Statistical analysis

All experiments were performed three times. Data presented as mean ± standard error of the mean (SEM). The data were analyzed with one-way ANOVA, with Bonferroni post hoc test for multiple comparisons. Differences were considered statistically significant at *p* value < 0.05 and indicated by‘*’, *p*<0.01 were indicated by ‘**’, *p*<0.001 were indicated by ‘***’. All the Scale bars in pictures represented 100 μm.

## Results

### Cells characterization

**Fig 1:**
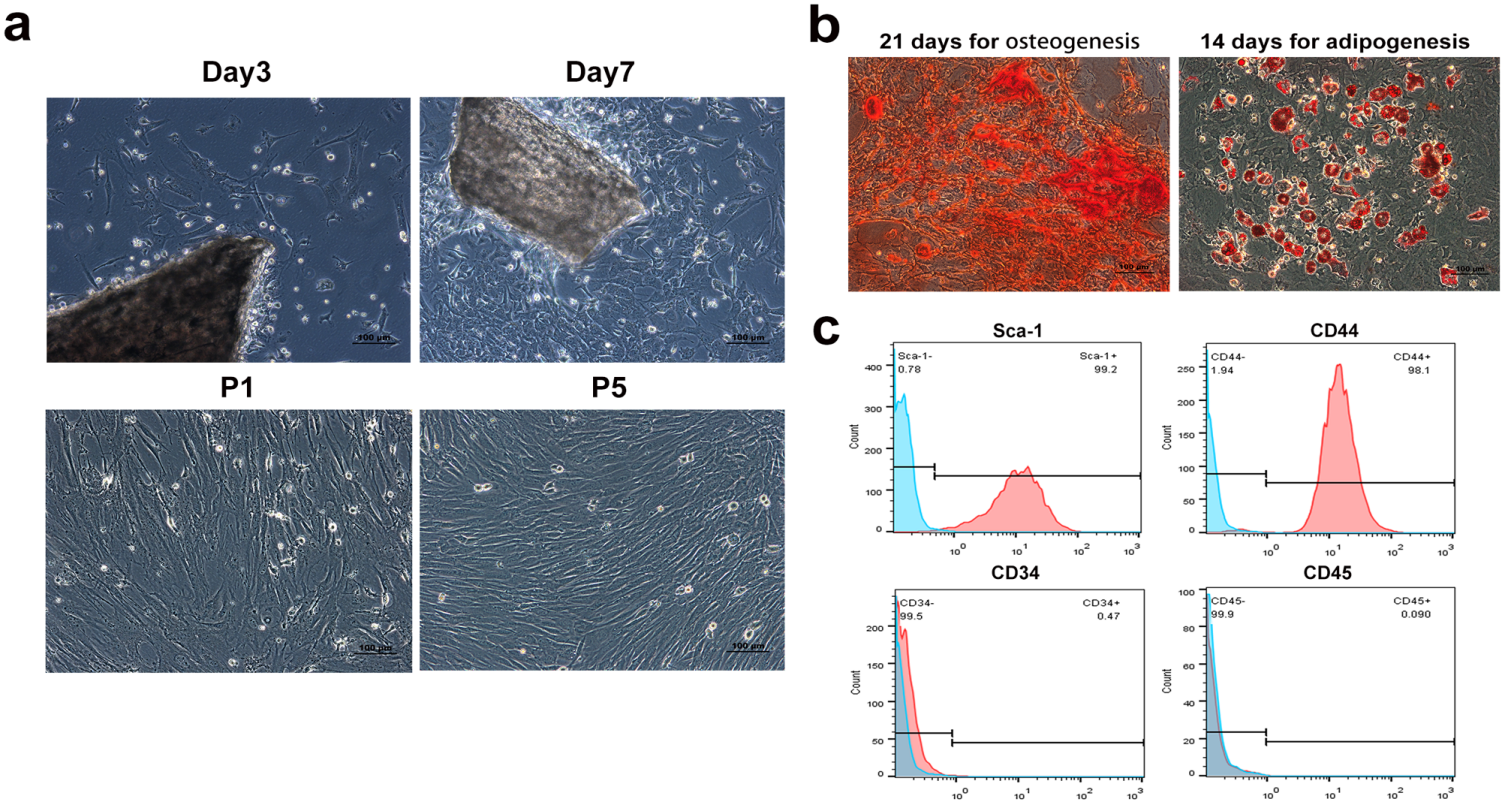
Characteristics and identification of compact bone-derived mouse mesenchymal stem cells. (a) Spindle cells migrated out from bone chips after a 3-d culture and reached 80–90% confluence after additional 4 days. A monolayer of homogeneous vortex-shaped cells were observed on passage 5. (b) Mineralized nodule were assayed by alizarin red staining and adipogenesis of MSC was stained with Oil red O. (c) Flow cytometric analysis showed that the cells were positive for Sca-1 and CD44 and negative for CD34 and CD45.

MSCs appeared as a monolayer of large, fibroblast-like flattened cells which were able to adopt an osteogenic or adipogenic phenotype under appropriate conditions (Fig 1a and 1b). Flow cytometric analysis showed that MSCs significantly expressed stem cell marker Sca-1 (97.8±0.4%) and CD44 (96. 0±1.6%), and were negative for hemopoietic stem cell marker CD34 (1.2±0.2%) and CD45 (0.3±0.1%) (Fig 1c).

**Fig 2:**
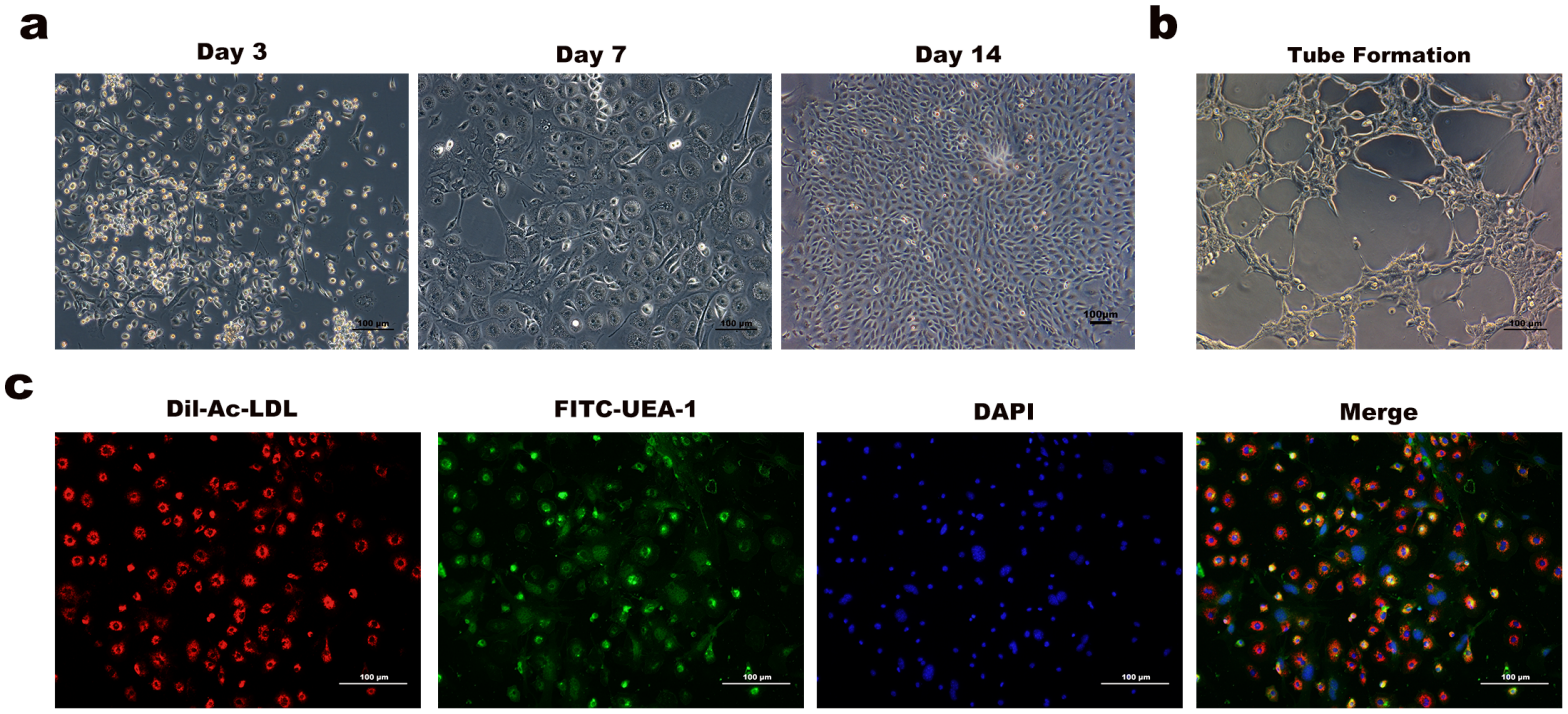
Characteristics and identification of cultured EPCs. (a) The cobblestone-like morphology of EPCs formed colonies after a 14d culture. (b) EPCs were cultured with EGM-2MV for 4h and formed capillary-like tubes on the ECM. (c) Immunofluorescence characterization showed that the majority of cells were capable of endocytosis both Dil-Ac-LDL and FITC-UEA-1.

The endothelial progenitor cells exhibited the cobblestone-like morphology after a 14-d culture (Fig 2a). Tube formation was observed under light photography, suggesting active angiogenic potency (Fig 2b). Immunofluorescence staining showed that majority of the attached cells were double positive for Dil-Ac-LDL and FITC-UEA-1, which were identified as differentiating EPCs (Fig 2c).

**Fig 3:**
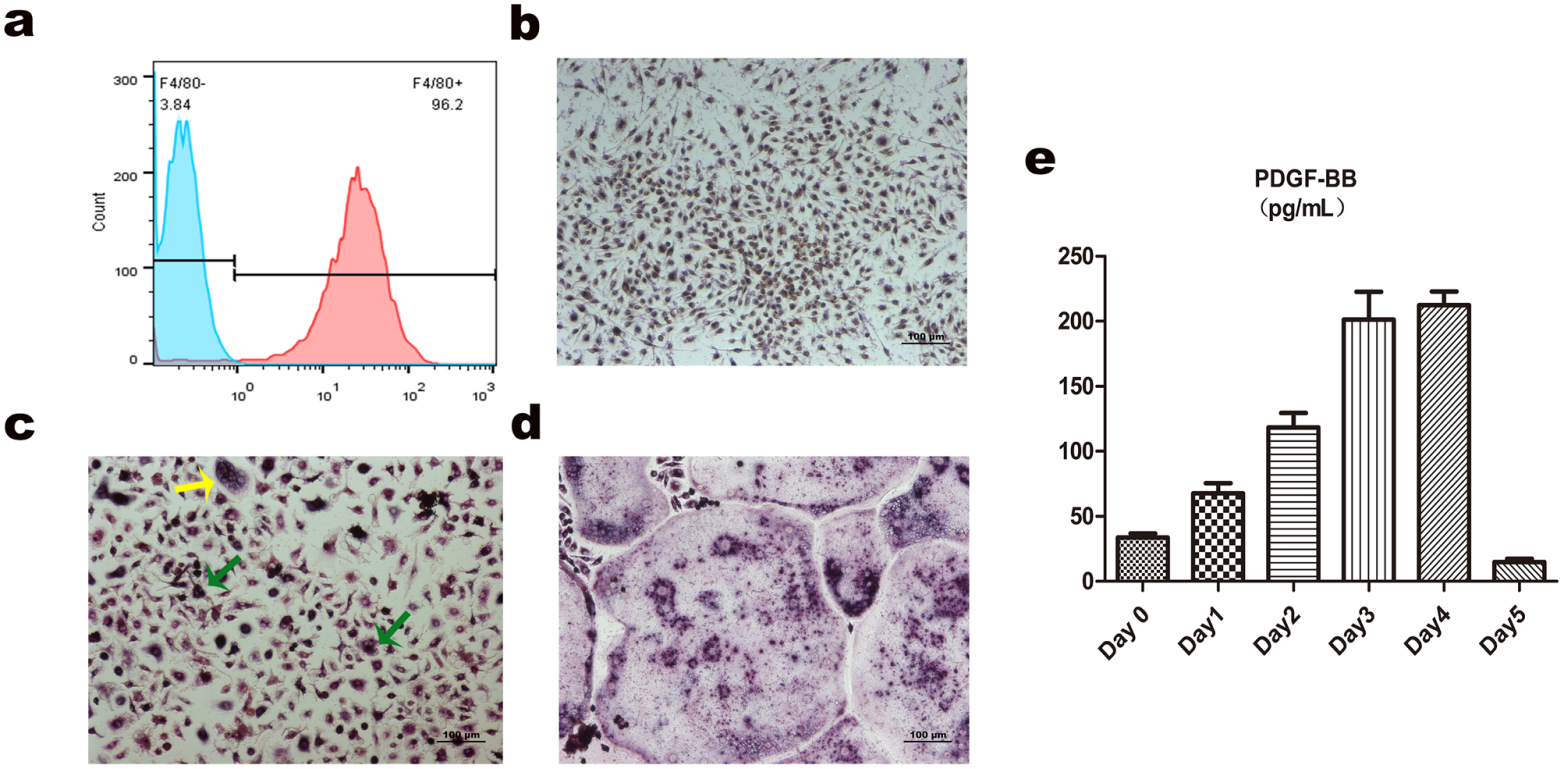
Identification of osteoclasts and secretion of PDGF-BB. (a) Flow cytometric analysis showed that adherent cells showed strong positive expression of F4/80. (b-d) TRAP staining during osteoclast formation. (b) Macrophages showed negative for TRAP staining. (c) After osteoclast differentiation for 72 h, almost all of cells became preosteoclasts (green arrows) and the others infused into multinucleated but not mature enough osteoclasts (yellow arrow). (d) Mature multinucleated cells were infused on the 5^th^ day. (e) Secretion of PDGF-BB by ELISA increased substantially and peaked during 72–96 h, along with the formation of preosteoclasts.

Most monocytes transferred to culture plates grew as adherent cells after 3-d culture with M-CSF. Flow cytometric analysis revealed that adherent cells showed strong positive expression of F4/80 (96.6±1.1%) (Fig 3a). TRAP staining showed that OPCs being induced for 0h were TRAP negative (Fig 3b). After a 3d culture with M-CSF and RANKL, almost all of OPCs became preosteoclasts (TRAP-positive and the number of nuclei ≤3) and the others infused into multinucleated but not mature enough osteoclasts (Fig 3c). Fully mature osteoclasts were observed after further incubation for 2 d (Fig 3d) [17].

### PDGF-BB secretion by preosteolcasts

Enzyme-linked immunosorbent assay (ELISA) showed that during osteoclastogenesis, PDGF-BB secretion increased substantially after 48 h and peaked during 72–96 h, along with the emergence of preosteoclasts. The secretion declined sharply on day 5 when osteoclasts matured (Fig 3e). These results indicated that it was preosteoclasts that released PDGF-BB, which was similar with the reports published before [13].

### ZOL showed a toxic effect on viability of OPCs at a lower level compared to EPCs and MSCs

**Fig 4:**
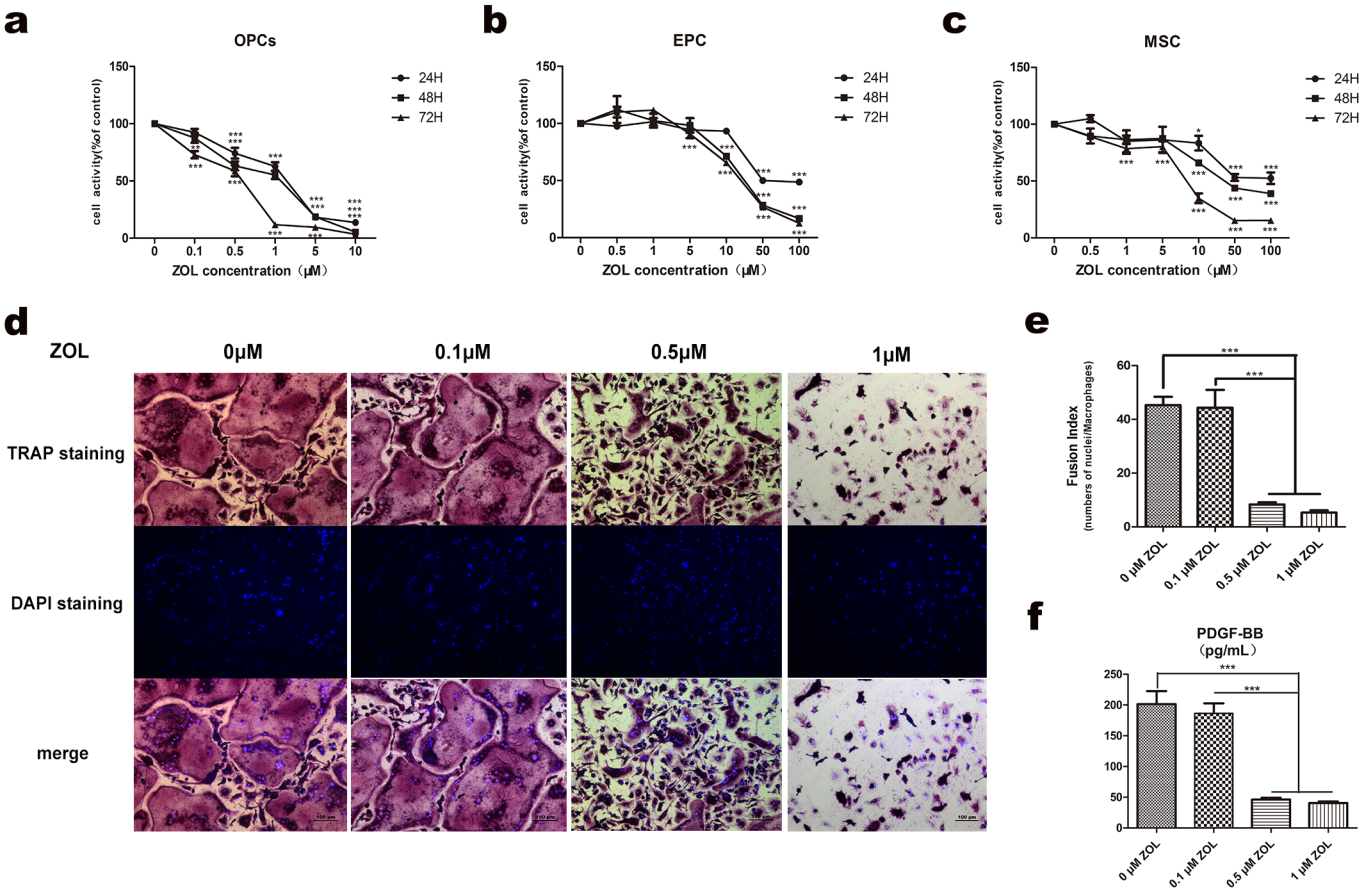
Zoledronate affected osteoclast formation and secretion of PDGF-BB. (a-c) Zoledronate affected viability of OPCs, MSCs and EPCs for 24, 48, 72 h. After a 48 h incubation, Zoledronate significantly inhibited the activity of OPCs at 0.1 μM (a) and showed obvious toxicity to EPCs and MSCs at 10 μM (b,c). (d) TRAP staining and DAPI counterstaining were made to show that zoledronate affected osteoclast formation. (e) Infusion index showed that zoledronate began to inhibit osteoclast formation obviously at 0.5 μM. (f) PDGF-BB secretion by ELISA also significantly decreased when treated by zoledronate at 0.5 μM for 48 h.

Osteoclast precursor cells cultured with M-CSF were treated with ZOL (0–10 μM) for 24, 48 and 72 h (Fig 4a). After 48 h incubation, ZOL inhibited viability of osteoclast precursors in a dose-dependent manner (*p*<0.01) and the IC50 value was 2.3 μM. EPCs and MSCs were treated with ZOL (0–100μM) for 24, 48 and 72 h. In order to compare with OPCs, as shown in Fig 4b and 4c, after 48 h incubation, ZOL significantly inhibited viability of EPCs at 10 μM (*p*<0.001) and MSCs at 10 μM (*p*<0.001). The IC50 value of EPCs was 22.2 μM and IC50 value of MSCs was 42.2 μM. The results showed that ZOL affected viability of OPCs prior to EPCs and MSCs at a lower concentration.

### ZOL inhibited osteoclast formation and secretion of PDGF-BB

According to the activity assay, OPCs were exposed to ZOL covering 0, 0.1, 0.5 and 1 μM for 2 days and then osteoclasts formation was tested by TRAP staining. Osteoclast precursor cells differentiated into TRAP positive multinucleated osteoclasts after a 5-d cultivation (Fig 4d). ZOL significantly inhibited osteoclast fusion at 0.5μM according to infusion index (45.3±5.5 vs 8.3±1.4, *p*<0.001) and caused apparent apoptosis at 1μM (Fig 4e).

Thus, we tested the serection of PDGF-BB by preosteoclasts on the 3^rd^ day, which were cultured with ZOL for the first 2 days. The results showed that when treated with 0.5μM ZOL, secretion of PDGF-BB decreased apparently (201.4±36.6 vs 46.0±5.1, *p*<0.001), in line with the osteoclast formation (Fig 4f).

### ZOL inhibited migration of EPCs and MSCs by PDGF-BB secretion

**Fig 5:**
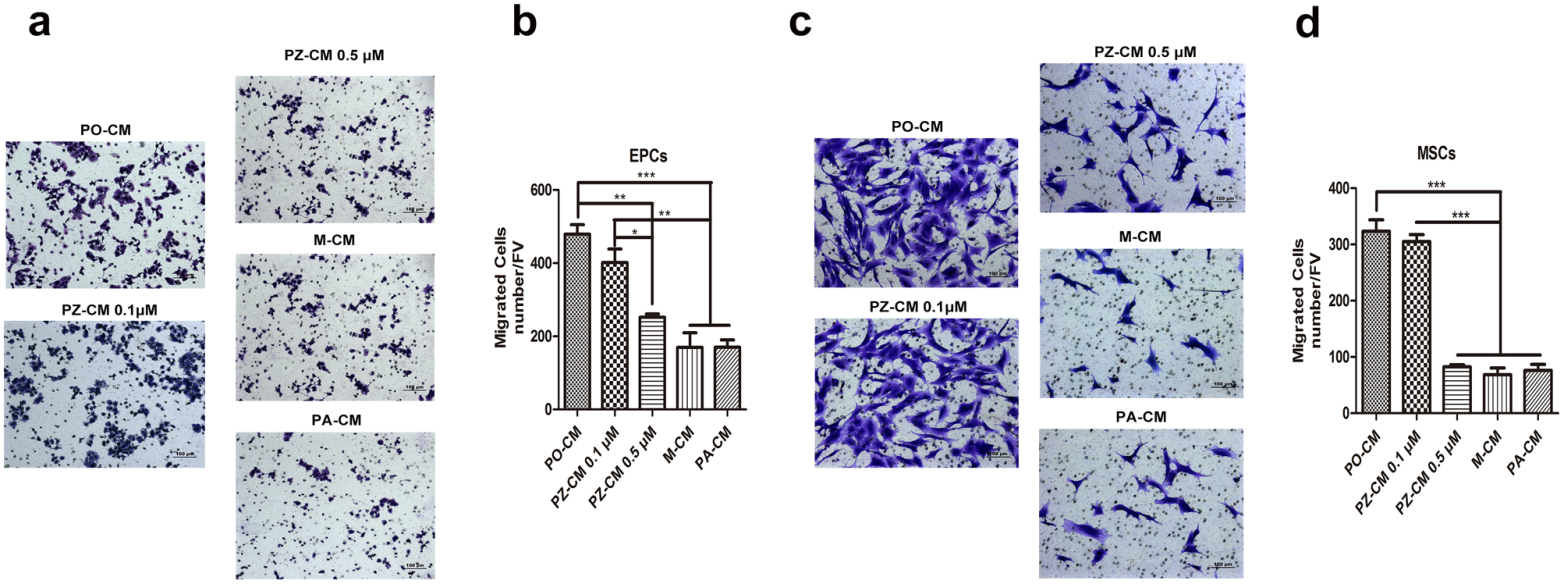
Different types of conditioned media affected the migration of EPCs and MSCs. (a, c) After 24 h, migrated EPCs and MSCs were stained by crystal violet. (b,d) Data analysis showed that conditioned media from preosteoclasts treated by zoledronate at 0.5 μM (PZ-CM 0.5μM) showed a significant decrease in the number of migrated cells compared with lower concentration and showed no difference with conditioned medium from macrophage (M-CM) or from preosteoclasts neutralized by PDGF-BB antibody (PA-CM).

In line with the concentration of PDGF-BB, as shown in Fig 5, we found that PO-CM significantly enhanced EPCs and MSCs migration, comparing with PZ-CM treated at 0.5 μM (479.0±45.3 vs 252.3±15.7, *p*<0.001 for EPCs and 323.3±35.6 vs 82.3±6.7, *p*<0.001 for MSCs). Besides, the PDGF-BB-free PA-CM lost the capacity of chemotaxis by comparison to PO-CM (*p*<0.001 for both kinds of cells). There were no statistical differences between the chemotaxis of PA-CM and M-CM or PZ-CM treated at 0.5μM (*p*>0.05).

This finding suggested that it was PDGF-BB that recruited EPCs and MSCs. ZOL could inhibit the migration of EPCs and MSCs through inhibiting osteoclast formation and secretion of PDGF-BB.

### ZOL inhibited PDGF-BB- inducing pro-angiogenesis of EPCs

**Fig 6:**
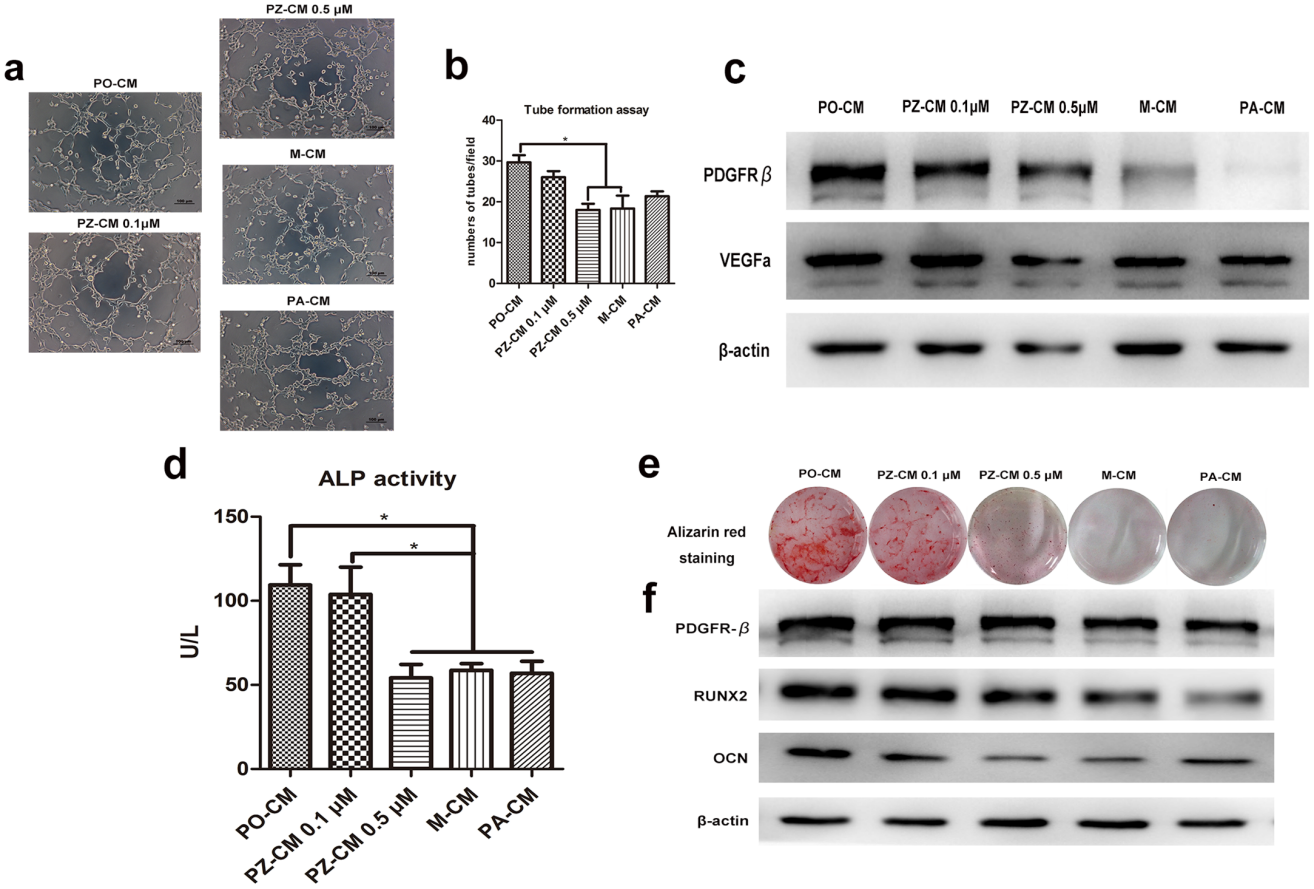
Different types of conditioned media affected angiogensis of EPCs and osteogenesis MSCs. (a) Conditioned media affected tube formation of EPCs. (b) Data analysis showed that PZ-CM 0.5 μM and M-CM but not PA-CM showed a weaker pro-angiogenic effect compared with PO-CM. (c) Western blot analysis of PDGFRβ and VEGFa in EPCs after culturing with conditioned media for 4 h. (d) ALP activity showed that MSCs co-culturing with PZ-CM 0.5 μM, M-CM or PA-CM had weaker tendency of osteogenesis. (e) Mineralized nodules were stained by Alizarin red S. (f) Western blot analysis of PDGFRβ, RUNX2 and OCN in EPCs after culturing with conditioned media for 48 h.

To determine whether the PDGF-BB promoted the angiogenesis of EPCs in vitro, we exposed EPCs to CM mentioned above for 4 h on Matrigel matrix. All CM induced EPC tube formation successfully (Fig 6a). The PO-CM promoted the tube formation of EPCs more evidently than M-CM (29.7±3.1 vs 18.3±5.5, *p*<0.05). Furthermore, conditioned medium from preosteoclasts treated by 0.5 μM ZOL reversed the pro-angiogenesis (18.0±2.7 vs 29.7±3.1, *p*<0.05) and there were no statistical difference in pro-angiogenic effect on EPCs between M-CM and PZ-CM treated at 0.5μM (*p*>0.05). Interestingly, PA-CM showed weaker function of tube formation compared to PO-CM but there was no statistical differences between them (29.7±3.1 vs 21.3±2.1, *p*>0.05) (Fig 6b).

### ZOL inhibited PDGF-BB inducing osteogenesis of MSCs

MSCs were cultured with CM for 2 days for ALP assay and cultured for 21 days for Alizarin Red S staining of matrix mineralization. Consistent with the above findings, as shown in Fig 6d, PO-CM significantly promoted ALP activity compared to M-CM /PA-CM (109.4±20.8 vs 58.7±6.9/52.4±14.7, *p*<0.05), which suggested that PDGF-BB secreted by preosteoclasts promoted osteogenic differentiation of MSCs. The tremendous decline of CM from preosteoclasts cultured with ZOL at 0.5 μM compared with PO-CM (54.1±13.9 vs 109.4±20.8, *p*<0.05) indicated that ZOL inhibited osteogenesis of MSCs, which was induced by PDGF-BB. At the same time, the results of the Alizarin Red S staining revealed a similar results at matrix mineralization formation (Fig 6e). The effect of PDGF-BB on the MSCs in our study was similar with previous research that recombination PDGF-BB was able to rescue the osteogenic capability of osteoblasts obtained from BRONJ patients [20].

### ZOL down-regulated bone-related gene expression of MSCs induced by PDGF-BB

Osteocalcin (OCN) is one of the very few osteoblast-specific proteins, defining molecular bases of bone physiology and Runt-related transcription factor 2 (RUXN2) is a key transcription factor, which is essential for osteoblastic differentiation and skeletal morphogenesis [21]. As shown in Fig 6f, when MSCs were co-cultured with PO-CM, the expression of OCN and RUNX2 of MSCs significantly increased comparing with CM-M or CM from preosteoclasts treated with PDGF-BB neutralizing antibody or ZOL at 0.5 μM. Besides, the expression of PDGFRβ of each group showed no significant difference.

### ZOL down-regulated VEGF expression of EPCs induced by PDGF-BB

VEGF is a remarkable single growth factor that regulates neovascularization such as vessel branching, maturation and quiescence so predominantly [22]. In our study, we tested EPCs the expression of VEGF and PDGFR-β, the receptor of PDGF-BB. Western blot assay showed that the expression of VEGF and PDGFR-β of EPCs both increased when cultured with PO-CM, comparing with M-CM or PA-CM. Conditioned medium from preosteoclasts treated by ZOL at 0.5 μM significantly down-regulated the expression of VEGF, as well as PDGF-BB, indicating that ZOL may down-regulated VEGF expression by PDGFR-β signaling axis (Fig 6c).

## Discussion

Bisphosphonate related osteonecrosis of the jaw (BRONJ) was a severe complication of systemic administration of bisphosphonate. First reported in 2003 by Marx, BRONJ happened to the patients exposing to the bisphosphonate in the treatment of osteoporosis and bone metastatic malignancies [1]. Although a few researchers tried to illustrate the mechanism of BRONJ, such as inhibition of angiogenesis, abnormality of inflammatory response and angiogenesis, inhibition of fibroblast etc, the exact mechanism of BRONJ is still unclear [5, 23–26].

Inhibition of angiogenesis was widely accepted as a mechanism of BRONJ, since the bisphosphonate was able to decrease the vessel density of jaw in vivo, and inhibit the endothelial cell from proliferation in vitro [24]. However, Denosumab, another antiresorptive medication which targeted the osteoclastogensis by blocking RANKL signal, was proved to have no effect on endothelial cell in vitro but to be able to decrease the vessel density in bone in patient with giant cell granuloma [27, 28]. Moreover, osteonecrosis or refractory osteomyelitis was commonly seen in patients with congenital osteopetrosis, which characterized by osteoclastogenesis deficiency [29]. Thus, deficiency in osteoclastogenesis might contribute to the occurrence of osteonecrosis of jaw.

Bone injury (tooth extraction) was a very important etiological factor of osteonecrosis of jaw. The injury can activate the osteoclastogenesis, which coupled the angiogenesis and osteogenesis [13, 30, 31]. Bisphosphates may inhibit the process of coupling of angiogenesis and osteogenesis by inhibiting osteoclastogenesis. Endothelial progenitor cells and marrow mesenchymal cells took significant role in the angiogenesis-osteogenesis coupling process. In our study, ZOL at 0.5μM was sufficient to inhibit osteoclasts formation and the chemotaxis of EPCs by condition medium from preosteoclasts. What was more, direct suppression of angiogenesis of EPCs or osteogenesis of MSCs by ZOL could be achieved at a much higher concentration, up to 50 folds comparing to osteoclastogenesis suppression (The results were not shown in the article but in S1 Fig). Thus, the anti-angiogenic effect of bisphosphonate in vivo might be primarily caused by the inhibition of EPC recruitment during osteoclasts formation, rather than direct effect on of endothelial cell. Then the insufficient vessel formation influenced recruitment of MSCs and its environment for osteogenic differentiation. Sufficient angiogenesis was important to resist the bacterial invasion from the site of tooth extraction, as well. If the process of osteoclastogenesis was interfered, the jaw bone remodeling, angiogenesis and osteogenesis might be suppressed. This might be a reasonable explanation to the in vivo angiogenic inhibition in status of osteoclastogenesis deficiency, which might contribute to the occurrence of osteonecrosis of jaw.

The PDGF family, consisting of PDGF-AA, PDGF-AB, and PDGF-BB, is well known for vessel maturation, and PDGF-BB was especially well documented in contributing to vascular repair/remodeling in humans and animal models after vascular injury [32]. Xie et al [13] revealed that PDGF-BB played the key cytokine of facilitating blood vessel growth and invasion of osteoprogenitor cells during bone development. What was more, it was also demonstrated that although endothelial progenitor cells or the rest bone marrow cells could release PDGF-BB, bone marrow PDGF-BB was primarily from preosteoclasts [33, 34]. Hence, in this study we sought to determine what role PDGF-BB played in zoledronate affecting the angiogenesis and osteogenesis. Osteoclastogenesis inducing PDGF-BB secretion significantly promoted the migration of EPCs and MSCs, which can be suppressed by ZOL. Along the cytokine gradient by PDGF-BB, zoledronate affected in vitro tube formation function of EPCs and osteoblastic differentiation of MSCs. Further western blot confirmed that the VEGF expression of EPCs down-regulated accompanied with PDGFR-β expression when cultured with PZ-CM, indicating that the PDGF-BB-PDGFR-β signaling between preosteoclasts and EPCs was important of bisphosphonate induced indirect angiogenic inhibition. The expression of RUNX2 and OCN of MSCs also showed positive correlation with the secretion of PDGF-BB in conditioned medium. There was no obvious difference of PDGFR-β expression of MSCs between groups might because the change of PDGFR-β expression was instant, which cannot be detected two days later.

It is worth mentioning that compared to the conditioned medium from preosteoclasts, the PDGF-BB antibody treated conditioned medium did not significantly weaken the capacity of in vitro tube formation of EPCs. This reminded us that there may be some other cytokines or exosome induced pro-angiogenesis of EPCs. And the inhibition of EPCs recruitment by PDGF-BB antibody might result from the insufficiency of PDGFR-β, which made itself sensitive to other cytokines or exosome. And another thing to note is that we found ZOL showed significant toxicity to OPCs at 0.1uM and inhibited osteoclast formation at 0.5 μM. It seemed that the inhibition of osteoclasts formation and PDGF-BB secretion mainly resulted from significant reduction in viability of OPCs, which is different from the former research [15] and further experiments is need to settle our question.

In conclusion, the inhibition of osteoclastogenesis-induced-coupling of angiogenesis and osteogenesis via PDGF-BB-PDGFR-β signaling by zolendonates, which was important in bone remodeling and healing, might contribute to the pathogenesis of BRONJ. It may be instructive to prevent or treat patients suffering from BRONJ by some simple and feasible methods. And further experiments are needed to support our hypothesis, such as PDGF-BB local treatment in vivo animal models.

## Supporting information

S1 Fig ZOL inhibited angiogenesis of EPCs and osteogenesis of MSCs. (a) Tube formation assay of EPCs treated by different concentrations of ZOL for 48 h. (b) Data analysis showed that ZOL at 10 μM significantly inhibited angiogenesis of EPCs. (c) ALP assay showed that a ZOL-exposure for 48 h at 10 μM significantly inhibited osteogenesis of MSCs.
(TIF)
