# Supplementary figures and images for: Zoledronate suppressed angiogenesis and osteogenesis by inhibiting osteoclasts formation and secretion of PDGF-BB
Source: PLoS One. 2017 Jun 8;12(6):e0179248. doi: 10.1371/journal.pone.0179248 (PMC5464661; doi:10.1371/journal.pone.0179248)

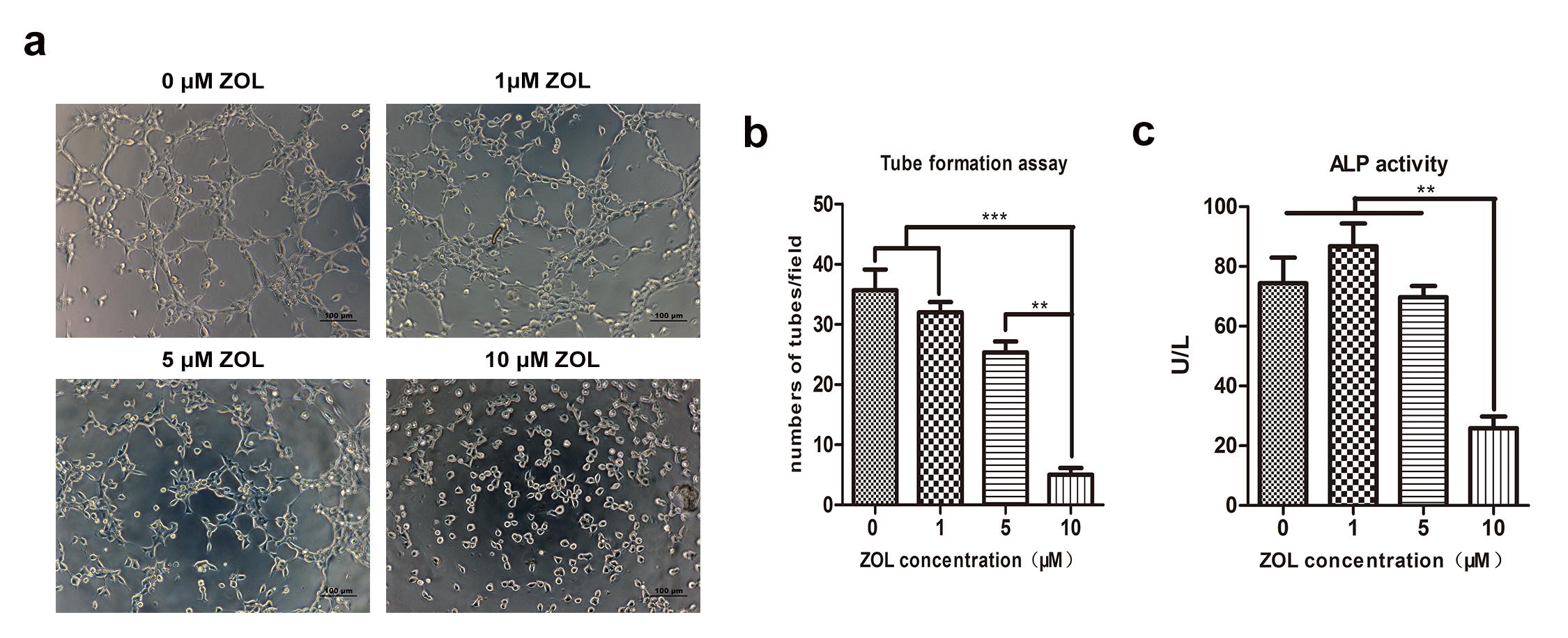

Supplement: S1 Fig — (a) Tube formation assay of EPCs treated by different concentrations of ZOL for 48 h. (b) Data analysis showed that ZOL at 10 μM significantly inhibited angiogenesis of EPCs. (c) ALP assay showed that a ZOL-exposure for 48 h at 10 μM significantly inhibited osteogenesis of MSCs. (TIF) [file pone.0179248.s001.tif]
